# ﻿Two new species of *Sinopoda* from China, with first description of the male of *S.horizontalis* Zhong, Cao & Liu, 2017 (Araneae, Sparassidae)

**DOI:** 10.3897/zookeys.1159.101535

**Published:** 2023-05-02

**Authors:** Jianshuang Zhang, Yuanqian Xing, Jinghui Yang, Hao Yu, Yang Zhong

**Affiliations:** 1 The State Key Laboratory of Southwest Karst Mountain Biodiversity Conservation of Forestry, Administration, School of life sciences, Guizhou Normal University, Guiyang, China Guizhou Normal University Guiyang China; 2 School of Biological Sciences, Guizhou Education University, Guiyang, Guizhou, China Guizhou Education University Guiyang China; 3 School of Nuclear Technology and Chemistry & Biology, Hubei University of Science and Technology, Xianning, Hubei, China Hubei University of Science and Technology Xianning China; 4 Administrative Commission of Jiugongshan National Nature Reserve of Hubei Xianning, Xianning, Hubei, China Administrative Commission of Jiugongshan National Nature Reserve of Hubei Xianning Xianning China

**Keywords:** Huntsman spider, morphology, new species, southern China, taxonomy

## Abstract

Three species of spider genus *Sinopoda* Jäger, 1999 are reported from southern China. Two of them are described as new to science: *S.guiyang* Zhang, Yu & Zhong, **sp. nov.** and *S.xishui* Zhang, Yu & Zhong, **sp. nov.**, both from Guizhou Province. The male of *S.horizontalis* Zhong, Cao & Liu, 2017 is described for the first time based on new material from the type locality, Wuyishan National Nature Reserve, Fujian Province, China. Detailed descriptions, diagnoses, photographs and a distribution map of the three species are provided.

## ﻿Introduction

*Sinopoda* Jäger, 1999 is the fourth most species-rich genus of huntsman spider family Sparassidae after *Pseudopoda* Jäger, 2000 (251 species), *Heteropoda* Latreille, 1804 (189 species), and *Olios* Walckenaer, 1837 (166 species) (World Spider Catalog 2023; Zhang et al. 2023), including 139 species so far. *Sinopoda* is mainly distributed in eastern Asia, with 86 species recorded from East Asia, 50 species reported from Southeast Asia, and a single species described from India (Zhang et al. 2021; Zhong et al. 2022). Currently, a total of 73 *Sinopoda* species are known from China, representing 52.5% of the global species, making China the country with the most *Sinopoda* species (Zhang et al. 2021; Zhong et al. 2022; World Spider Catalog 2023). Despite this fact, the diversity of this genus in China is still not fully discovered as several new species have been described in each of the last few years (Zhong et al. 2017, 2018, 2019, 2022; Grall and Jäger 2020; Zhu et al. 2020; Wang et al. 2021).

While examining spiders collected from southern China, we have found some *Sinopoda* specimens that belong to three species: two of them from Guizhou Province, belong to undescribed species new to science; the remaining one from Wuyishan National Nature Reserve of Fujian Province, was identified as *Sinopodahorizontalis* Zhong, Cao & Liu, 2017 based on comparison with the type specimens (previously described based on a holotype female only). The goal of this paper is to describe the two new species under the names of *S.guiyang* Zhang, Yu & Zhong, sp. nov. and *S.xishui* Zhang, Yu & Zhong, sp. nov. and to redescribe *S.horizontalis*, describing the male for the first time.

## ﻿Materials and methods

Specimens in this study were collected by hand. The type specimens are deposited in the Museum of Guizhou Normal University, Guiyang, China. Specimens were preserved in 75 or 95% alcohol and examined using an Olympus SZX7 stereomicroscope. Left male palps were examined and illustrated after dissection. Epigynes were removed and cleared in a warm 10% potassium hydroxide (KOH) solution. The vulva was imaged after being embedded in Arabic gum. Images were captured with a Canon EOS 70D digital camera (20.2 megapixels) mounted on an Olympus CX41 compound microscope, and assembled using Helicon Focus ver. 6.80 image stacking software. All measurements were obtained using an Olympus SZX7 stereomicroscope and are given in millimetres. Eye diameters were measured at the widest part. The total body length does not include the chelicerae or spinnerets. Leg lengths are given as total length (femur, patella + tibia, metatarsus, tarsus). Numbers of macrosetae are listed for each segment in the following order: prolateral, dorsal, retrolateral and ventral (in femora and patellae ventral spines are absent and fourth digit is omitted in the setation formula). The distribution map was generated with ArcGIS ver. 10.5 (Environmental Systems Research Institute, Inc.). The terminology used in the text and figure legends follows Grall and Jäger (2020) and Zhong et al. (2019, 2022), and the abbreviations used in the text or figures are given in Table 1.

**Table 1.** List of abbreviations used in the text or figures.

**Male palp**	
**C** = conductor	**EA** = embolic apophysis
**Cy** = cymbium	**EB** = embolic base
**RTA** = retrolateral tibial apophysis	**Sp** = spermophor
**dRTA** = dorsal branch of RTA	**St** = subtegulum
**vRTA** = ventral branch of RTA	**T** = tegulum
**E** = embolus	
**Epigyne**	
**AB** = anterior band	**LL** = lateral lobe
**FD** = fertilization duct	**LS** = lobal septum
**GA** = glandular appendage	**MS** = membranous sac
**ID** = internal duct	**PP** = posterior part of spermathecae
**Ocular area**	
**AER** = anterior eye row	**CH** = clypeus height
**ALE** = anterior lateral eye	**PER** = posterior eye row
**AME** = anterior median eye	**PLE** = posterior lateral eye
**AME–ALE** = distance between AME and ALE	**PME** = posterior median eye
**AME–AME** = distance between AMEs	**PME–PLE** = distance between PME and PLE
**AME–PLE** = distance between AME and PLE	**PME–PME** = distance between PMEs
**AME–PME** = distance between AME and PME	

## ﻿Taxonomy

**Family Sparassidae Bertkau, 1872**

### ﻿Subfamily Heteropodinae Thorell, 1873

#### ﻿Genus Sinopoda

Taxon classificationAnimaliaAraneaeSparassidae

Jäger, 1999

E6507653-248C-54C0-8205-66A79F905FFD

##### Type species.

*Sarotesforcipatus* Karsch, 1881 from China and Japan.

##### Diagnosis.

See Jäger (1999), Liu et al. (2008), Zhang et al. (2015) and Grall and Jäger (2020).

##### Composition and infrageneric groupings.

See WSC (2023) and Zhang et al. (2021).

#### ﻿ Sinopoda guiyang

Taxon classificationAnimaliaAraneaeSparassidae

Zhang, Yu & Zhong sp. nov.

7B655AA0-C616-5B5A-8B0A-4961A21ED05F

https://zoobank.org/425C8C5A-625C-4344-A882-373C7D5F440B

Figs 1 , 2 , 3 , 9

##### Type material.

***Holotype*** ♂ (YHSPA001), China: Guizhou Province: Guiyang City: Xinpu Town, Xiangzhigou, Nanjing temple, 26.75°N, 106.93°E, c. 1092 m, by hand, 14.VI.2017, H. Yu et al. leg. ***Paratypes***: 2♂3♀ (YHSPA002–006), same data as holotype.

##### Etymology.

The species name is derived from the name of the type locality; noun in apposition.

##### Diagnosis.

**Figure 1.**
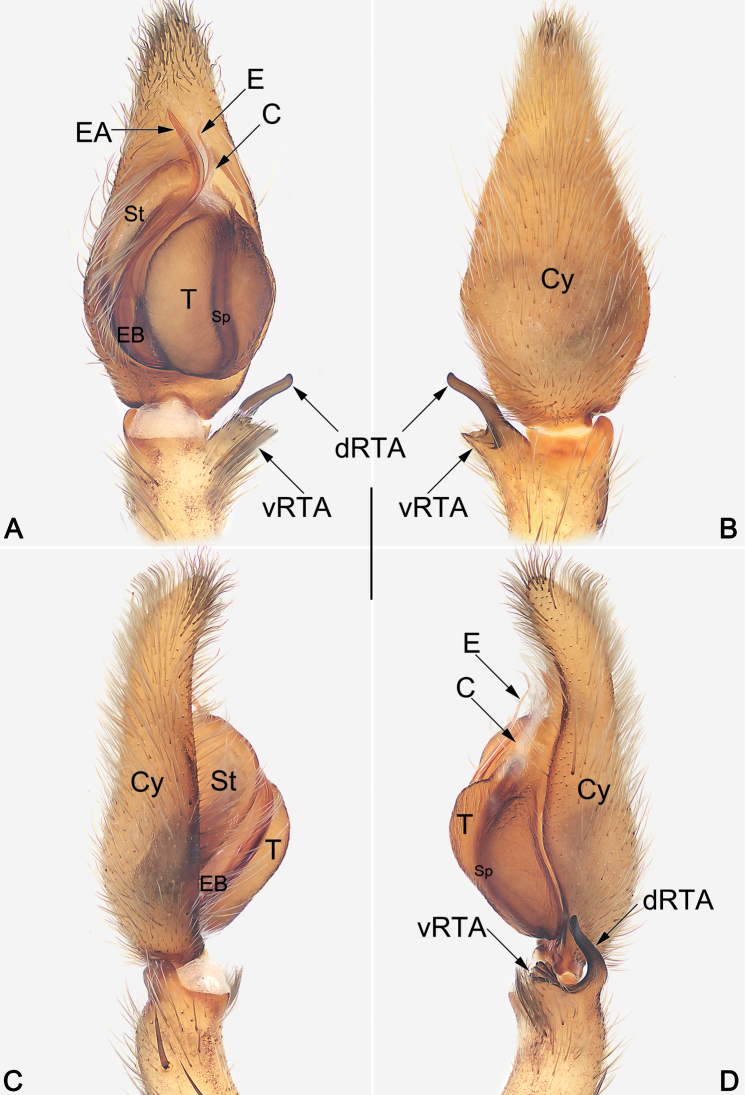
Male palp of the holotype of *Sinopodaguiyang* sp. nov. **A** ventral view **B** dorsal view **C** prolateral view **D** retrolateral view. Abbreviations: C = conductor; Cy = cymbium; dRTA = dorsal branch of RTA; E = embolus; EA = embolic apophysis; EB = embolic base; Sp = spermophor; St = subtegulum; T = tegulum; vRTA = ventral branch of RTA. Scale bar: 0.5 mm (equal for **A–D**).

**Figure 2.**
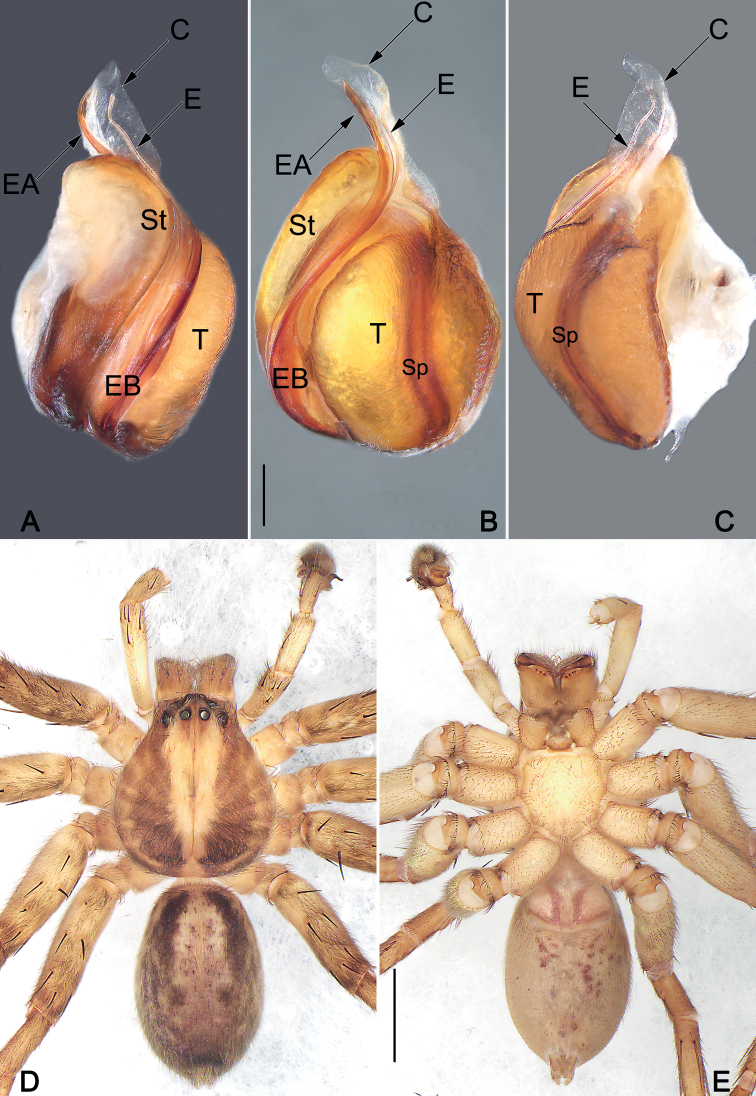
*Sinopodaguiyang* sp. nov., male holotype, palpal bulb (**A–C**) and habitus (**D, E**) **A** prolateral view **B** ventral view **C** retrolateral view **D** dorsal view **E** ventral view. Abbreviations: C = conductor; E = embolus; EA = embolic apophysis; EB = embolic base; Sp = spermophor; St = subtegulum; T = tegulum. Scale bars: 0.2 mm (equal for **A–C**); 2 mm (equal for **D, E**).

**Figure 3.**
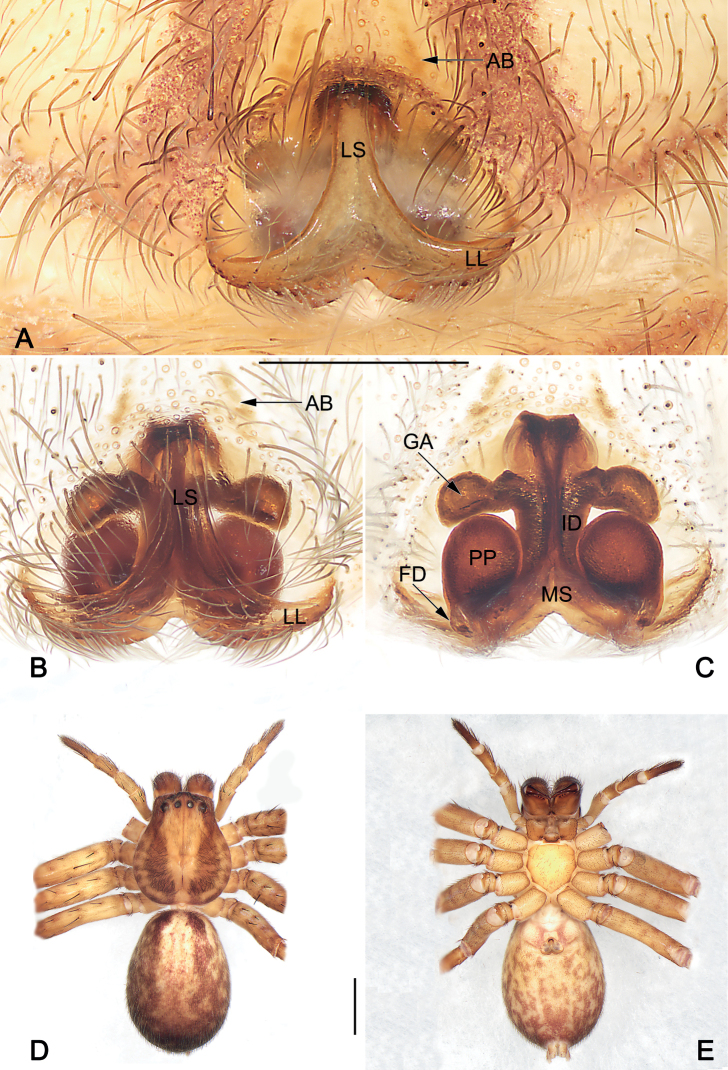
*Sinopodaguiyang* sp. nov., female paratype, epigyne (**A–C**) and habitus (**D, E**) **A** intact, ventral view **B** cleared and macerated, ventral view **C** cleared and macerated, dorsal view **D** dorsal view **E** ventral view. AB = anterior band; FD = fertilization duct; GA = glandular appendage; ID = internal duct; LL = lateral lobe; LS = lobal septum; MS = membranous sac; PP = posterior part of spermathecae. Scale bars: 0.5 mm (equal for **A–C**); 2 mm (equal for **D, E**).

The males of new species resemble those of *S.ovata* Zhong, Jäger, Chen & Liu, 2019 and *S.triangula* Liu, Li & Jäger, 2008 in having a short vRTA with rough apex, and a long, finger-like dRTA (Fig. 1A, B, D; Zhong et al. 2019: figs 43B, C, 44B, C; Liu et al. 2008: fig. 7B, C), but differ by: (1) subdistal embolus without triangular projection (vs. with a triangular projection) (cf. Figs 1A, D, 2A–C and Zhong et al. 2019: figs 43A, B, 44A, B and Liu et al. 2008: fig. 7A, B, D–F); (2) embolus whip-like or filiform, distinctly thin (vs. relatively thicker, distally wide) (cf. Figs 1A, D, 2A–C and Zhong et al. 2019: figs 43A, B, 44A, B and Liu et al. 2008: fig. 7A, B, D–F); (3) apex of vRTA with four ridges (vs. without ridges) (cf. Fig. 1D and Zhong et al. 2019: figs 43C, 44C and Liu et al. 2008: fig. 7C). Females also resemble those of *S.ovata* and *S.triangula* by the general shape of vulva but can be recognized by the thumb-like glandular appendages extend transversally (vs. finger-like and descend obliquely) (cf. Fig. 3C and Zhong et al. 2019: figs 43E, 45B and Liu et al. 2008: fig. 7H).

##### Description.

**Male (YHSPA001).** Total length 8.4. Prosoma 4.0 long, 3.4 wide, anterior width of prosoma 2.6. Opisthosoma 4.4 long, 2.6 wide. ***Eye sizes and interdistances***: AME 0.18, ALE 0.26, PME 0.18, PLE 0.27, AME–AME 0.19, AME–ALE 0.09, PME–PME 0.24, PME–PLE 0.35, AME–PME 0.33, ALE–PLE 0.28, CHAME 0.21, CHALE 0.23. ***Spination***: Palp: 131, 101, 1021; Fe: I–III 323, IV 321; Pa: I–IV 101; Ti: I 2024, II–III 2126, IV 2226; Mt: I–II 2024, III–IV 3036. ***Measurements of palp and legs***: Palp 6.3 (2.2, 1.3, 1.1, 1.7), I 15.5 (3.8, 1.9, 4.4, 4.0, 1.4), II 17.5 (4.8, 1.9, 4.7, 4.5, 1.6), III 14.1 (4.4, 1.5, 3.6, 3.3, 1.3), IV 15.4 (4.4, 1.7, 3.6, 4.1, 1.6). Leg formula: II-I-IV-III. Cheliceral furrow with three anterior and four posterior teeth, and with ~ 35 denticles.

***Colouration in ethanol* (Fig. 2D, E).** Prosoma yellowish-brown, anteriorly and medially yellowish, lateral and posterior margin dark brown, with shallow fovea and radial furrows. Chelicerae light brown. Sternum yellowish-white, margin yellowish. Endites and labium uniformly yellowish-white. Legs dark yellowish-brown, covered by short spines. Opisthosoma oval, dorsum brown, marginally with two longitudinal and dark brown bands reaching posterior half, median part with four pairs of inconspicuous purplish dots; venter uniformly gray.

***Palp* (Figs 1, 2A–C).** Cymbium distinctly longer than tibia. Embolus filiform, Ƨ-shaped in ventral view, arising at approximately the 8–9 o’clock position, terminating at c. 12 o’clock position. Conductor long, membranous, c. 2/3 of the tegulum length, originating at 12–1 o’clock position portion of tegulum. Tegulum oval, slightly bulged, medially with distinct and slightly curved spermophore, proximally covering embolic base. RTA arising mesially to distally from tibia, ventrally with distinct brush of stiff setae. dRTA slender, finger-shaped; vRTA round, apex with four ridges.

**Female (YHSPA002).** Total length 10.3. Prosoma 4.2 long, 3.6 wide, anterior width of prosoma 2.8. Opisthosoma 6.1 long, 4.6 wide. ***Eye sizes and interdistances***: AME 0.17, ALE 0.24, PME 0.20, PLE 0.29, AME–AME 0.21, AME–ALE 0.10, PME–PME 0.27, PME–PLE 0.39, AME–PME 0.39, ALE–PLE 0.34, CHAME 0.23, CHALE 0.26. ***Spination***: Palp: 131, 101, 2026, 1014; Fe: I–III 323, IV 321; Pa: I–IV 000; Ti: I–III 2026, IV 2126; Mt: I–II 1014, III 2026, IV 3036. ***Measurements of palp and legs***: Palp 5.0 (1.5, 0.9, 1.0, 1.6), I 12.2 (3.3, 1.5, 3.0, 3.2, 1.2), II 12.7 (3.8, 1.8, 3.2, 2.8, 1.1), III 10.3 (3.0, 1.6, 2.7, 2.1, 0.9), IV 11.9 (3.5, 1.7, 2.9, 2.7, 1.1). Leg formula: II-I-IV-III. Cheliceral furrow with three anterior and four posterior teeth, and with ~ 42 denticles. Colouration in ethanol as in males, but generally slightly darker (Fig. 3D, E).

***Copulatory organ* (Fig. 3A–C).** Epigynal field wider than long, with short and indistinct anterior bands, slit sensillum absent. Lobal septum wide, anterior part about 1/10 width of epigynal plate, gradually wider to the posterior. Lateral lobes fused, with small median incision and posterior margin slightly bilobed. Internal ducts running parallel along median line. Glandular appendages thumb-like, extend transversally. Posterior part of spermathecae balloon-shaped, relatively large, c. 1.6 times longer than wide; the two PP separated by about 0.8 width. Fertilization ducts acicular, membranous, located on dorsal-basal surface of spermathecae. Membranous sac between fertilization ducts, more or less triangular.

##### Distribution.

Known only from the type locality, Guiyang City, Guizhou, China (Fig. 9).

##### Comments.

*Sinopodaguiyang* sp. nov. possesses several characters associated with the *globosa*-group (currently comprises six species, see Zhang et al. 2021: 15, fig. 4) and resembles *S.ovata* and *S.triangula* (the core species of the *globosa*-group) for their characteristic genital organs (for a detailed diagnosis, see above), but can be distinguished from all members of the *globosa*-group by the absence of triangular projection in the embolus. Because the embolus of all *S.globosa*-group species has a subdistally triangular projection, there remains considerable uncertainty about placing this new species into the *globosa*-group.

**Figure 4.**
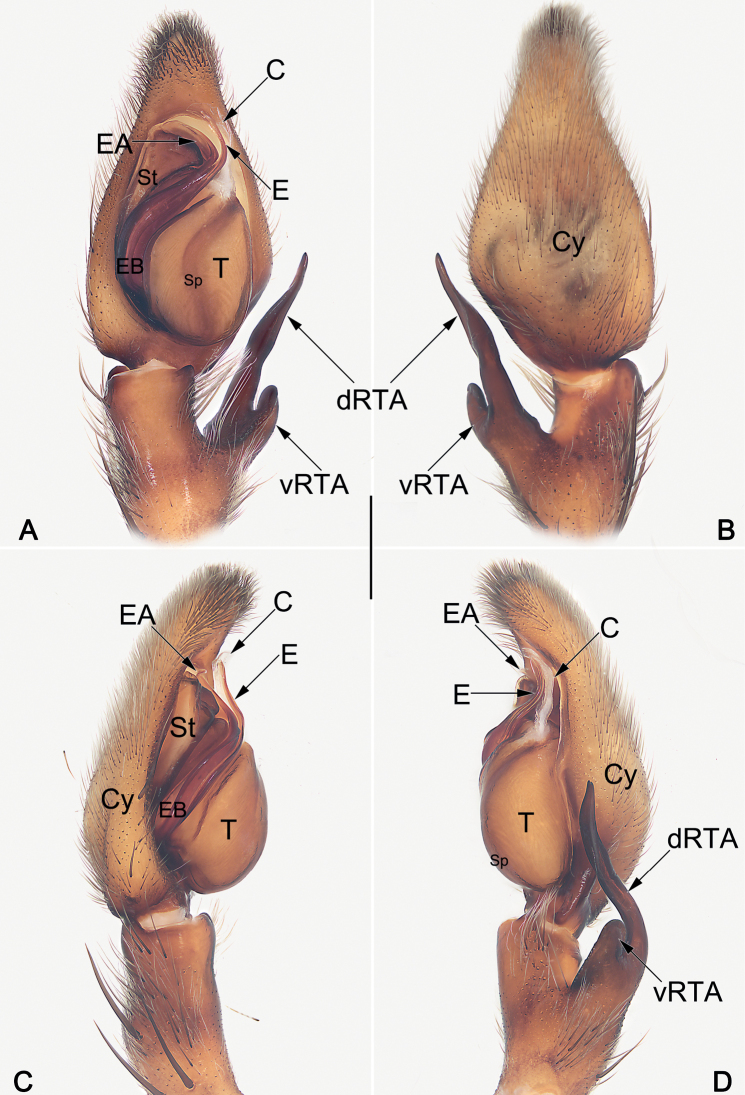
Male palp of the topotype of *Sinopodahorizontalis***A** ventral view **B** dorsal view **C** prolateral view **D** retrolateral view. Abbreviations: C = conductor; Cy = cymbium; dRTA = dorsal branch of RTA; E = embolus; EA = embolic apophysis; EB = embolic base; Sp = spermophor; St = subtegulum; T = tegulum; vRTA = ventral branch of RTA. Scale bar: 1 mm (equal for **A–D**).

#### ﻿ Sinopoda horizontalis

Taxon classificationAnimaliaAraneaeSparassidae

Zhong, Cao & Liu, 2017

719FAB83-3084-5101-9A95-D693A78E29F2

Figs 4 , 5 , 6 , 9

Sinopoda horizontalis Zhong, Cao & Liu, 2017: 157, figs 5A, B, 6A, B, 15A, B (♀).

##### Holotype examined.

♀ (ZY-2013-SPA007), China: Fujian Province: Wuyishan City, Wuyishan National Nature Reserve, 27.35°N, 117.29°E, c. 1152 m, by hand, 16 VI 2013, Y. Zhong and X.W. Cao leg.

##### New material examined.

3♂, 1♀ (ZY-2021-SPA011–014). Same locality as holotype, by hand, 16.VI.2021, Y. Zhong leg.

##### Diagnosis.

Males of *S.horizontalis* resemble those of *S.hamata* (Fox, 1937) and *S.liui* Zhong, Cao & Liu, 2017 in the general shape of the male palp. The palps of the three species share the similarly shaped conductor and embolus, and the distinctly long, ribbon-shaped dRTA which with lumpy margins, but differ in the following: the vRTA digitiform, distinctly longer than wide, apex blunt in *S.horizontalis* (vs. laminar, distinctly wider than long in *S.hamata*, thumb-shaped, apex beak-shaped and relatively sharper in *S.liui*) (cf. Fig. 4A, B, D and Zhong et al. 2018: figs 6C, 7C and Zhong et al. 2019: figs 31C, 32C). Females also resemble those of *S.hamata* and *S.liui* in having the strongly narrow lobal septum anteriorly, and the distinctly bilobed posterior margin of epigynal plate, but can be recognised from *S.hamata* by the internal ducts running parallel along median line (vs. convergent anteriorly but distinctly oblique posteriorly) (cf. Fig. 6C–F and Zhong et al. 2018: figs 6D, E, 7D, E); and from *S.liui* can be recognised by the posterior part of spermathecae are proportionately longer, nearly 2/5 length of internal ducts (vs. proportionately much shorter, no more than 1/4 length of internal ducts) (cf. Fig. 6C–F and Zhong et al. 2019: fig. 33B and Zhong et al. 2017: figs 5D, 6D).

##### Description.

**Male (ZY-2021-SPA011).** Total length 14.8. Prosoma 7.0 long, 6.7 wide, anterior width of prosoma 3.4. Opisthosoma 7.8 long, 5.2 wide. ***Eye sizes and interdistances***: AME 0.24, ALE 0.45, PME 0.22, PLE 0.50, AME–AME 0.21, AME–ALE 0.13, PME–PME 0.41, PME–PLE 0.38, AME–PME 0.48, ALE–PLE 0.40, CHAME 0.29, CHALE 0.35. ***Spination***: Palp: 131, 101, 1021; Fe: I–III 323, IV 321; Pa: I–IV 101; Ti: I–IV 2226; Mt: I–II 1014, III 2026, IV 3036. ***Measurements of palp and legs***: Palp 9.7 (2.8, 2.0, 2.1, 2.8), I 34.0 (9.9, 3.8, 8.4, 8.9, 3.0), II 35.8 (10.6, 3.8, 9.0, 9.5, 2.9), III 27.7 (7.8, 3.6, 7.2, 7.0, 2.1), IV 28.9 (8.6, 3.1, 7.0, 7.7, 2.5). Leg formula: II-I-IV-III. Cheliceral furrow with three anterior and four posterior teeth, and with ~ 32 denticles.

**Figure 5.**
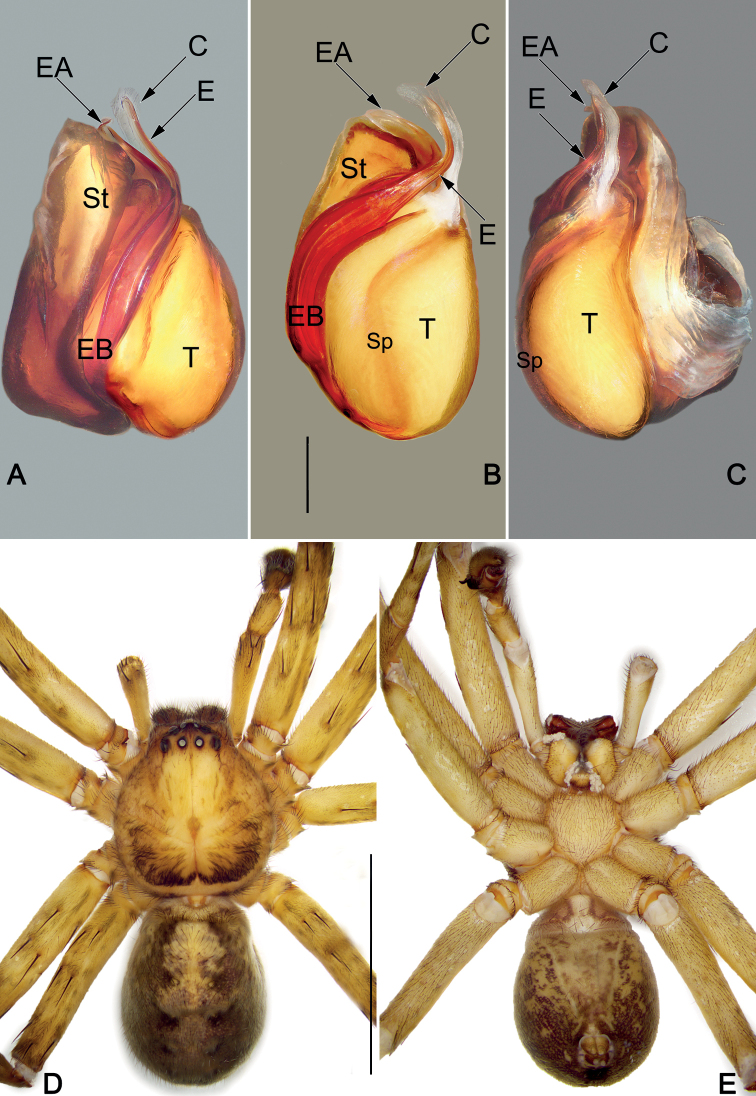
*Sinopodahorizontalis*, male topotype, palpal bulb (**A–C**) and habitus (**D, E**) **A** prolateral view **B** ventral view **C** retrolateral view **D** dorsal view **E** ventral view. Abbreviations: C = conductor; E = embolus; EA = embolic apophysis; EB = embolic base; Sp = spermophor; St = subtegulum; T = tegulum. Scale bars: 0.2 mm (equal for **A–C**); 5 mm (equal for **D, E**).

***Colouration in ethanol* (Fig. 5D, E).** Prosoma dark yellowish to brown, with yellow submarginal transversal band at posterior part. Median band of prosoma bright yellowish-brown, lateral bands brown and not distinctly delimited to median band. Fovea and radial furrows distinctly marked. Chelicerae dark reddish-brown. Sternum light yellow, margin slightly darker. Endites and labium yellowish, both with distal parts brighter. Legs dark yellowish-brown, covered by short spines. Dorsal opisthosoma brown, with an irregular yellow media band, reaching 2/3 of abdomen length, with five pairs of inconspicuous black dots on each side; ventral opisthosoma yellowish-brown with irregular pattern and two longitudinal yellow lines between epigastric furrow and spinnerets.

***Palp* (Figs 4, 5A–C).** Cymbium distinctly longer than tibia. Embolus filiform, Ƨ-shaped, arising from tegulum at nearly the 7–8 o’clock position in ventral view, terminating at c. 12 o’clock position. Conductor long, c. 2/3 of the tegulum length, curving medially, arising at 12- 1o’clock position from tegulum. Tegulum oval, slightly bulged, medially with distinct spermophore, proximally covering embolic base; spermophore <-shaped in ventral view. RTA arising mesially to distally from tibia, ventrally with distinct brush of stiff setae. dRTA ribbon-shaped, distinctly long, curved and tapering, almost extending media part of cymbium; vRTA digitiform, relatively short, about 1/2 of tibia length, apex round.

**Figure 6.**
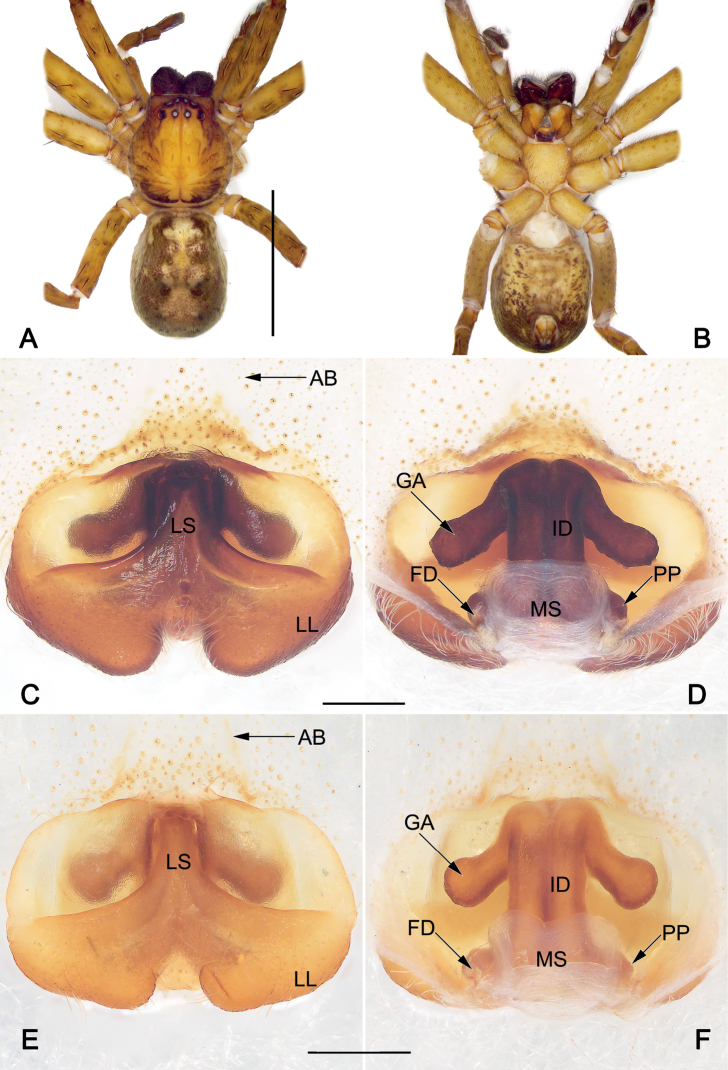
*Sinopodahorizontalis*, habitus (**A, B**) and epigyne (**C, D**) of female topotype, and epigyne (**E, F**) of female holotype **A** dorsal view **B** ventral view **C, E** cleared and macerated, ventral view **D, F** cleared and macerated, dorsal view. AB = anterior band; FD = fertilization duct; GA = glandular appendage; ID = internal duct; LL = lateral lobe; LS = lobal septum; MS = membranous sac; PP = posterior part of spermathecae. Scale bars: 5 mm (equal for **A, B**); 0.5 mm (equal for **C, D**, equal for **E, F**).

**Female (ZY-2021-SPA014).** Total length 14.9. Prosoma 7.6 long, 7.3 wide, anterior width of prosoma 4.5. Opisthosoma 9.3 long, 5.8 wide. ***Eye sizes and interdistances***: AME 0.30, ALE 0.48, PME 0.36, PLE 0.58, AME–AME 0.25, AME–ALE 0.17, PME–PME 0.45, PME–PLE 0.61, AME–PME 0.62, ALE–PLE 0.66, CHAME 0.31, CHALE 0.45. ***Spination***: Palp: 131, 101, 2121, 1014; Fe: I–III 323, IV 321; Pa: I–IV 101; Ti: I–III 2024, IV 2124; Mt: I–II 1014, III 2026, IV 3036. ***Measurements of palp and legs***: Palp 9.6 (2.8, 1.4, 2.2, 3.2), I 24.3 (6.3, 2.6, 6.3, 6.6, 2.5), II 25.9 (8.3, 3.3, 7.1, 5.2, 2.0), III 20.2 (7.7, 3.4, 3.6, 3.8, 1.7), IV 22.2 (7.1, 2.5, 5.8, 4.9, 1.9). Leg formula: II-IV-III-I. Cheliceral furrow with three anterior and four posterior teeth, and with ~ 40 denticles. Colouration in ethanol as in males, but body slightly darker (Fig. 6A, B; see Zhong et al. (2017) for others described). Copulatory organ as in Fig. 6C, D (topotype) and Fig. 6E, F (holotype).

##### Distribution.

Known only from the type locality, Wuyishan National Nature Reserve, Fujian, China (Fig. 9).

#### ﻿ Sinopoda xishui

Taxon classificationAnimaliaAraneaeSparassidae

Zhang, Yu & Zhong sp. nov.

F22BB1C1-F238-549F-B90F-2E73588A3F50

https://zoobank.org/75A10745-DBF5-4845-905C-D2CB6DE32005

Figs 7 , 8 , 9

##### Type material.

***Holotype*** ♀ (YHSPA007), China: Guizhou Province: Zunyi City: Xishui County, Xishui National Nature Reserve, Sanchahe Town, Hongyangou Village, 28.50°N, 106.40°E, c. 934 m, by hand, 23.V.2022, H. Yu et al. leg. ***Paratype***: 1♀, same data as holotype.

##### Etymology.

The species name is derived from the name of the type locality; noun in apposition.

##### Diagnosis.

**Figure 7.**
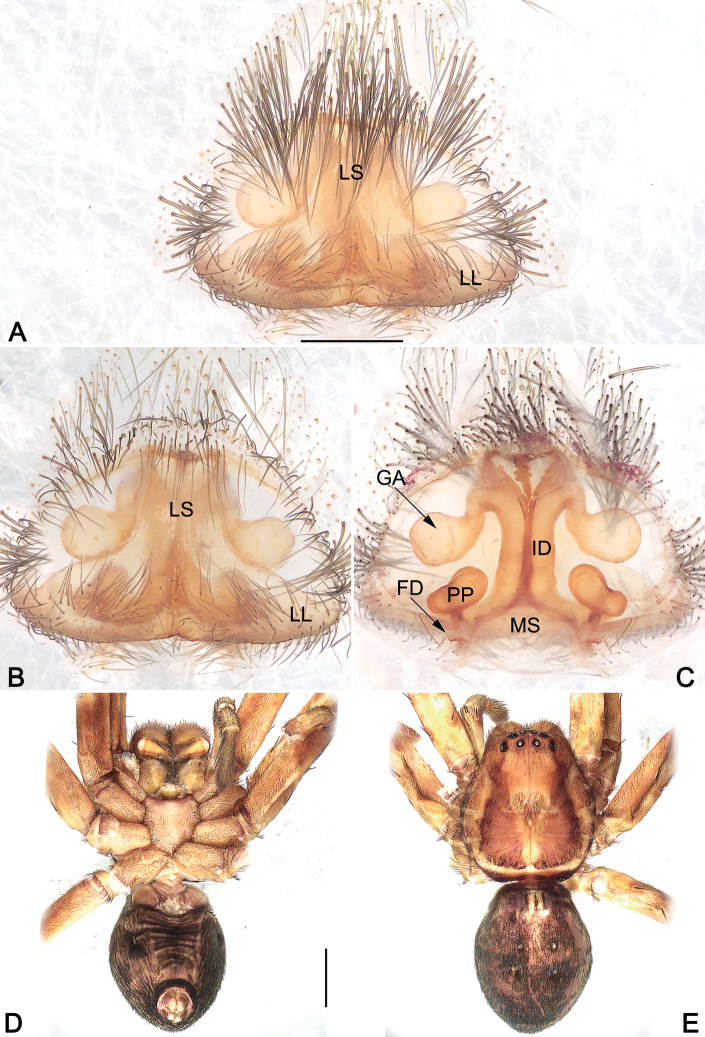
*Sinopodaxishui* sp. nov., female holotype, epigyne (**A–C**) and habitus (**D, E**) **A** macerated, ventral view **B** cleared and macerated, ventral view **C** cleared and macerated, dorsal view **D** dorsal view **E** ventral view. FD = fertilization duct; GA = glandular appendage; ID = internal duct; LL = lateral lobe; LS = lobal septum; MS = membranous sac; PP = posterior part of spermathecae. Scale bars: 0.5 mm (equal for **A–C**); 3 mm (equal for **D, E**).

Females of this new species resembles those of *S.yaanensis* Zhong, Jäger, Chen & Liu, 2019 in having similar vulva with swollen, globular glandular appendages, and oval shaped posterior part of spermathecae, but can be distinguished by: (1) lobal septum distinctly wider, its anterior part about 1/5 width of epigynal plate (Fig. 7A, B) (vs. relatively narrower, its anterior part about 1/8–1/9 width of epigynal plate; Zhang et al. 2015: figs 40, 46; Zhong et al. 2019: fig. 57E); and (2) the anterior part of internal ducts far from the anterior margin of epigynal plate (Fig. 7C) (vs. reach the anterior margin of epigynal plate; Zhang et al. 2015: fig. 41; Zhong et al. 2019: fig. 57J).

##### Description.

**Female (YHSPA007).** Total length 16.4. Prosoma 7.7 long, 6.8 wide, anterior width of prosoma 4.2. Opisthosoma 8.7 long, 6.1 wide. ***Eye sizes and interdistances***: AME 0.35, ALE 0.44, PME 0.38, PLE 0.48, AME–AME 0.28, AME–ALE 0.14, PME–PME 0.44, PME–PLE 0.53, AME–PME 0.52, ALE–PLE 1.44, CHAME 0.32, CHALE 0.37. ***Spination***: Palp: 131, 101, 2121, 1014; Fe: I–III 323, IV 321; Pa: I–IV 101; Ti: I–III 2026, IV 2126; Mt: I–II 1014, III 2026, IV 3036. ***Measurements of palp and legs***: Palp 9.8 (3.0, 1.6, 2.1, 3.1), I 26.8 (7.8, 2.4, 7.8, 6.5, 2.3), II 29.1 (8.9, 2.8, 8.2, 7.1, 2.1), III 24.2 (7.6, 3.1, 6.3, 5.1, 2.1), IV 24.7 (6.7, 2.4, 7.3, 6.1, 2.2). Leg formula: II-I-IV-III. Cheliceral furrow with two anterior and four posterior teeth, and with ~ 38 denticles.

**Figure 8.**
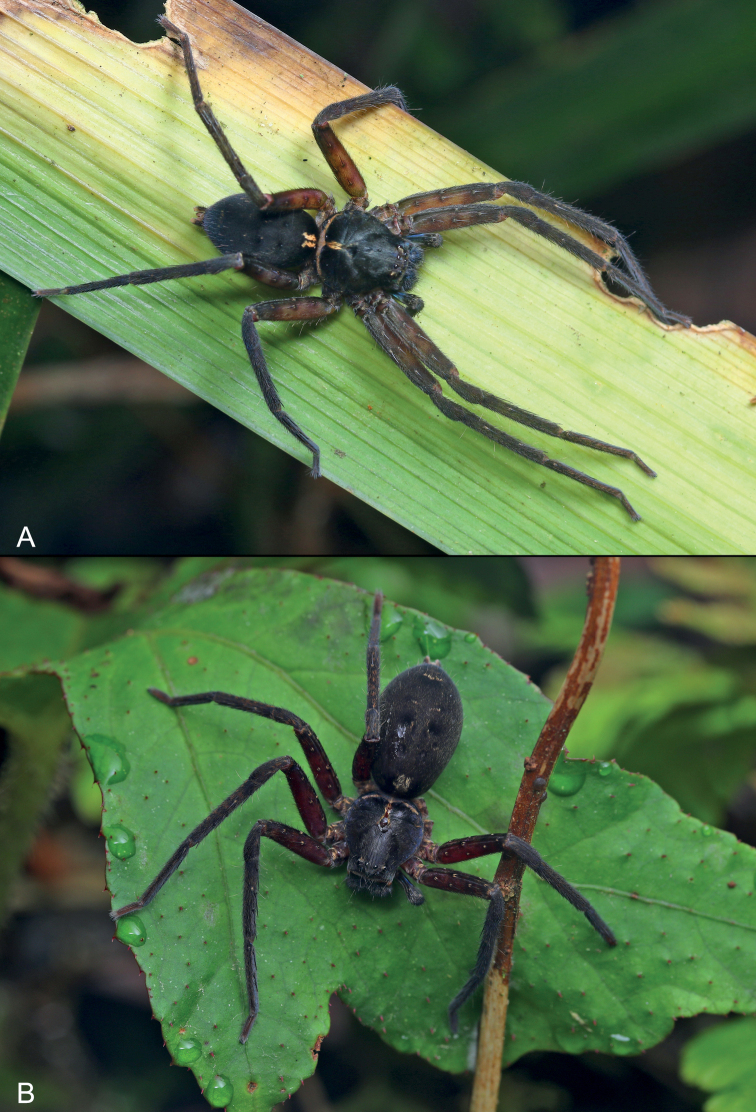
*Sinopodaxishui* sp. nov., female holotype (**A**) and paratype (**B**), live specimens. Photographs by Qianle Lu (Shenzhen, Guangdong).

Colour of the living holotype female was uniformly dark except brown femur (Fig. 8A). Colouration in ethanol (Fig. 7D, E): Prosoma dark yellowish to brown, with bright yellow submarginal transversal band at posterior part. Median band of prosoma bright yellowish, anteriorly as wide as PER, gradually narrowing posteriorly; lateral bands brown, distinctly delimited to median band, starting from PLE, reaching dark reddish submarginal transversal band. Fovea and radial furrows distinctly marked. Chelicerae yellowish-brown. Sternum bright yellow, margin slightly darker. Endites and labium yellowish. Legs yellowish-brown, covered by short spines. Dorsal opisthosoma dark brown, anteriorly with a small ‘)(‘-shaped yellow pattern, with three pairs of inconspicuous dots on each side; ventral opisthosoma dark, with several transversal folds.

***Copulatory organ* (Fig. 7A–C).** Epigynal field wider than long, anterior bands nearly invisible indistinct, slit sensillum absent. Lobal septum wide, anterior part about 1/5 width of epigynal plate, gradually wider to the posterior. Lateral lobes fused, posterior margin slightly bilobed, medially with small incision. Internal ducts running parallel along the middle line. Glandular appendages distinctly inflated, globular; the two GA widely separated by about 3× diameters. Posterior part of spermathecae more or less bean-shaped, c. 1.9 times longer than wide; the two PP separated by about 2.3 width. Fertilization ducts acicular, membranous, located on posterior surface of spermathecae. Membranous sac between fertilization ducts, nearly trapezoidal.

**Male.** Unknown.

##### Distribution.

**Figure 9.**
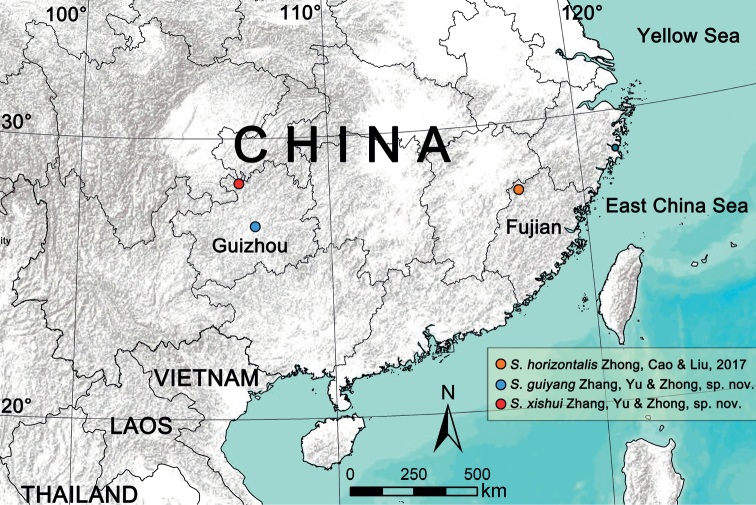
Distribution records of the *Sinopoda* species treated in this paper: *S.horizontalis* Zhong, Cao & Liu, 2017 (orange circle: Fujian Province: Wuyishan City, Wuyishan National Nature Reserve), *S.guiyang* sp. nov. (blue circle: Guizhou Province, Guiyang City, Xinpu Town, Xiangzhigou), *S.xishui* sp. nov. (red circle: Guizhou Province, Zunyi City, Xishui National Nature Reserve).

Known only from the type locality, Xishui National Nature Reserve, Guizhou, China (Fig. 9).

##### Comments.

The females of *S.xishui* sp. nov. exhibit typical *globosa*-group features (internal ducts running parallel along median line, and with ovate posterior parts of spermathecae, as diagnosed in Zhang et al. (2021)), and resembles *S.yaanensis* (the core species of the *globosa*-group) (for a detailed diagnosis, see above). However, this species is not readily assignable to the *globosa*-group due to the lack of an available male specimen.

## Supplementary Material

XML Treatment for Sinopoda

XML Treatment for Sinopoda guiyang

XML Treatment for Sinopoda horizontalis

XML Treatment for Sinopoda xishui
